# Efficacy of swarm-based neural networks in automated depression detection

**DOI:** 10.1038/s41598-025-09414-z

**Published:** 2025-07-15

**Authors:** Alwan Atta, Dina ElSayad, Doaa Ezzat, Safaa Amin, Mahmoud ElGamal

**Affiliations:** 1 Scientific Computing Department, Faculty of Computers and Information Sciences, Ain Shams University, Cairo, Egypt; 2 Galala University, Suez, Egypt

**Keywords:** Mental disorders, Neural network, Dragonfly algorithm, Firefly algorithm, Moth flame optimization, Diagnosis, Clinical trial design, Diseases, Psychiatric disorders, Trauma

## Abstract

As depression becomes a global pandemic, this research paper presents a comprehensive study for depression diagnosis using a custom-crafted deep learning model optimized with various swarm intelligence algorithms. Three different optimization algorithms-dragonfly algorithm, Firefly Algorithm, and Moth Flame Optimization Algorithm-have been employed for feature selection and dimensionality reduction. The experiments were done with the DAIC-WOZ corpus, which is a benchmark dataset for depression detection. In the first experiment, the dragonfly algorithm was used and gave a macro F1 score 0.76, recall of 0.80, and precision of 0.74. The second experiment was the implementation of the Firefly Algorithm, which outperformed the first with a macro F1 score of 0.86, recall of 0.88, and precision of 0.85. The third experiment based on the Moth Flame Optimization Algorithm achieved a macro F1 score of 0.80, a recall rate of 0.79, and a precision of 0.72. Next, the models were put through two additional datasets to test how well they generalize. Therefore, the model achieved 0.92 F1 on the CMDC dataset and 0.82 on the MODMA dataset, establishing strong performance across different distributions of data. The results highlight how the integration of deep learning techniques with metaheuristic optimization algorithms can provide optimal and reliable depression diagnosis and thus create promising directions for further research in this area.

## Introduction

Major mental disorders are considered to be an important public health concern around the world. According to the World Health Organization, one in every eight people in the world lives with a mental disorder^1^. An estimated 22.8% of adults in the United States experienced mental illness in 2021, which corresponded to 57.8 million people. This includes many disorders such as anxiety disorders, bipolar disorder, schizophrenia, and major depressive disorder. The prevalence rate for these conditions simply brings to the fore the dire need for effective diagnosis, treatment, and management modalities. Of the myriad mental health disorders, depression is prominent because of its relatively high prevalence and the depth with which it might strike an individual’s life. Major depressive disorder (MDD) affects an estimated 8.3% of adults in the United States each year. Depression^2^ is a mood disorder characterized by severely degraded mood, loss of interest in activities that used to be enjoyed, and several physical and cognitive symptoms that are severe enough to interfere with everyday functioning. Although relatively common, it is generally rated among clinicians as one of the most notoriously awkward diagnoses to make. Thus, there exist a lot of impediments related to the diagnosis process itself. First of all is the heterogeneity issue; not only can depression develop because of a great multitude of elements, but symptoms and types alone vary from person to person. According to DSM-5, 227 symptoms can combine in all, as criteria for MDD^3^. This makes diagnosis very difficult because two patients with depression may not share a single symptom in common. Another complication arises because the symptoms are subjective: many patients may underreport or overreport symptoms due to stigma, lack of awareness, or difficulty in describing their experience^4^. Comorbid conditions, in particular anxiety substance use disorders, and chronic medical illnesses, complicate the clinical presentation, making the diagnosis of major depression as the principal problem complicated. These challenges bring along with them an increased exploration toward more innovative methodologies that improve the precision and dependability of diagnoses of depression. Over the past years, several varieties of models and techniques have been put forward incorporating various advanced technologies. Nickson et al.^5^ explored the role of machine learning algorithms in the prediction and diagnosis of depression by analyzing electronic health records. Indeed, these studies unveiled the quite good performance of the machine-learning-based models-essentially regression algorithms in the classification tasks, with the AUC very often far above 0.85. Squires et al.^6^ carried out a broad systematic review related to deep learning and machine learning in psychiatric practice. These studies proved that, if tapped properly, these technologies have immense potential to implement precision psychiatry-applying approaches that are more individualized in the screening, diagnosis, and treatment of depression. It also brought out the need for empirical validation by randomized controlled trials laying down a model to achieve better results in patient outcomes. Also, several studies^7^ discussed how the integration of these heterogeneous data sources could improve depression detection. It aims at capturing subtle and multi-dimensional features of depressive symptoms that might lead to an accurate diagnosis^8^. Advanced computational techniques are leading to an evolving landscape of mental health diagnosis, in particular regarding depression. During such difficulties, particularly presented by the traditional approaches toward diagnosing depression because of its heterogeneous and subjective nature, promising innovative approaches come forward through machine learning and analysis of multimodal data. The continued need for research and empirical validation is necessary to translate these technological advancements into clinical practice and, therefore, to further enhance the accuracy of depression diagnosis and quality of care in affected individuals with this pervasive mental health condition.

## Related work

Recently, automatic depression diagnosis based on speech has received a large amount of attention with the advent of new neural network architecture^9^ and optimization techniques based on swarm intelligence. This paper presents a literature review^10^ on very recent research works that mainly focus on methodologies, types of data, and algorithms employed in this domain. The analysis has placed the challenges and developments in focus concerning speech data for depression detection, with emphasis on the role of neural networks and swarm intelligence in improving diagnosis effectiveness. Speech data is one of the very essential elements in the identification of depression due to its non-invasive nature and because it can convey much information about the emotional and mental state of a person. Various acoustic features, such as pitch, tone, and speech rate, can indicate depressive symptoms. Recent works have taken advantage of these features to develop systems that can diagnose depression quite well. A recent study by Zhang et al.^11^ used a large dataset of speech recordings to train Convolutional Neural Networks (CNNs) in detecting depression. In this respect, their model achieved a very high accuracy rate that presents the ability of speech data in the identification of depressive symptoms. Along similar lines, Lee et al.^12^ employed an RNN, where temporal patterns in speech were modeled, and performance was further improved. Neural networks and, most recently, deep learning models have indeed shown great promise in processing and analyzing speech data to detect depression. Such models can extract relevant features from a raw speech signal themselves and can diminish the tedium associated with manual feature engineering. CNNs can grasp the spatial hierarchies in the data, a capability that has made them very popular. In the speech data, CNNs are capable of detecting patterns in the acoustic features related to depression. Kim et al.^13^ have applied CNNs in detecting depression from speech recordings and reported a maximum accuracy of 85%. RNNs, including LSTMs, are naturally in the position of analyzing sequential data. The speech application will then capture the temporal dependencies that are key to understanding how depressive symptoms evolve. A study by Wang et al.^14^ utilized LSTM networks on speech data and recorded significant improvement in diagnostic accuracy as compared to traditional methods. Transformer models have also been adapted to speech data with their inherent attention mechanisms for depression detection. Such a model could focus on the most relevant parts of the speech signal, enhancing this model’s capability for detecting subtle changes associated with depression. Recently, Chen et al. showed that transformer models do exceptionally well in this domain, reaching state-of-the-art performance. Swarm intelligence has been generally inspired by how social creatures collaborate and has been applied to optimize neural network settings and improve model performances. Swarm intelligence has been generally inspired by how social creatures collaborate and has been applied to optimize neural network settings and improve model performances. The methods of Particle Swarm Optimization (PSO) and Ant Colony Optimization (ACO) have been applied to tune neural networks in the direction of detecting depression. PSO is an algorithm inspired by flying birds or shoaling fishes. It has also been applied to optimize the settings of neural networks to improve their performance. One recent work, by Liu et al.^15^, uses PSO to optimize a convolutional neural network that detects depression and improves its performance. intelligence technique is the ACO, inspired by the foraging behavior of ants; it has also been used to optimize neural networks. The study conducted by Gupta et al.^16^ illustrated the efficiency of ACO in optimizing Recurrent Neural Networks (RNNs) for speech-based depression detection and its superiority to traditional optimization methods. Along with all the advancements, there are a number of challenges that persist in the diagnosis of depression using automated systems with speech data. One huge challenge is the difference in the speeches amongst individuals and cultures, which makes the application of such a model to everybody very difficult. Moreover, speech can be recorded in different quality and consistency, introducing noise and usually affecting the performance of the models. Further research into strong model creation, which should perform well for a wide range of groups of people, forms part of the next stages in this field. Some of the useful techniques include Transfer Learning and Domain Adaptation in enhancing the model. The integration of facial expressions with physical signals seems quite encouraging regarding the accurate detection of depressive states. Several recent works present new approaches to further enhance the accuracy and reliability of the detection of depression by using speech-related input data, neural networks, and swarm intelligence. Among these, the work of Patel et al.^17^ presented a hybrid model consisting of CNNs and RNNs optimized by PSO for the detection of depression. In the hybrid model, the advantages of CNN and RNN were effectively exploited to accomplish a significant improvement in diagnostic accuracy. Another recent related work by Singh et al.^18^ presented the integration of speech data with other modalities, such as facial expressions and physiological signals, via a transformer-based model. Again, this is a case that demonstrates the improvement of this model for the detection of depression through diverse data source integration. Various explainable AI techniques have been applied to make the models more interpretable. A study by Zhao et al.^19^ applied XAI methods to identify the most relevant speech features for depression detection, thus providing insight into the underlying mechanisms of the models. The research on the application of transfer learning with wav2vec 2.0 to depression detection was also conducted by Xu Zhang et al.^20^ They fine-tuned wav2vec 2.0 with the purpose of extracting depression-related features and integrated 1D-CNN and attention pooling structures to enhance feature representation at the segment level. Du et al.^21^ explored transfer learning using wav2vec 2.0 for depression classification on low-resource setups. Their results have shown how pre-trained models make use of large volumes of untranscribed speech to substantially improve performance. The model proposed by the researchers combined the key features of speech production and perception, hence commanding an exponentially higher increase in the strides of improvement pertaining to the precision of detection. Du et al. discussed a speech chain model integrating speech production and perception features into a single framework for depression detection. This model easily isolated those minor features of speech that manifested a depressive condition, including aspects such as prosody, articulation, and acoustic patterns. Zhou et al.^22^ then proposed a hierarchical multi-feature fusion strategy in recognizing depression. The model contained an audio-response-level approach that combined a host of speech characteristics, further enhancing recognition. The results indeed seemed promising to describe the complex relationships among features of audio associated with depressive states.

Neural networks and swarm intelligence in the automated diagnosis of depression using speech data have a great deal of promise. Recent progress has shown how those technologies could enhance diagnostic accuracy and reliability. However, challenges persist, and future research should be directed at developing robust and generalizable models and integrating more multimodal data to increase the detection of depression. Continued innovation and empirical validation are needed to translate these advances into clinical practice in a way that will raise the quality of care for individuals affected by depression.

## Approach

In this section, we detail the methodologies employed to diagnose depression from speech data.

### Mel-frequency cepstral coefficients

**Fig. 1:**
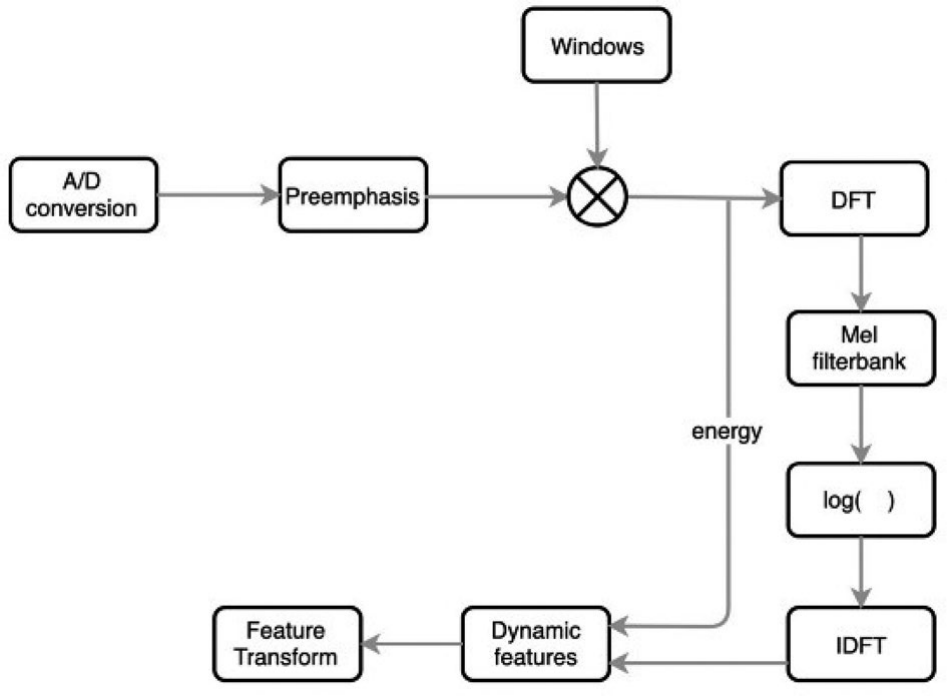
Mel-frequency cepstral coefficients framework^24^.

Mel-Frequency Cepstral Coefficients (MFCCs)^23^ are widely used in speech processing and feature extraction. The coefficients represent a short-term power spectrum of an acoustic signal, which is the result of a linear cosine transform of a logarithmic power spectrum calculated on a nonlinear Mel-frequency scale. MFCCs work as follows: The audio signal is segmented into overlapping frames to accurately represent the temporal dynamics of speech. Fourier transform is applied to each frame for the conversion of the time-domain signal into the frequency domain. Then power spectrum mapping onto the Mel scale is done through a filter bank. The Mel scale approximates the response of the human ear more closely than the linear frequency axis. The power in each Mel-frequency is logarithmically transformed to model the loudness perception of the human auditory system. Discrete Cosine Transformation (DCT) is applied to the log-Mel spectrum, which would uncorrelate the coefficients and reduce dimensionality. The MFCCs are the coefficients obtained after this step, and Fig. 1 shows the steps illustrated. MFCCs are imperative in speech recognition applications and speaker identification by describing the features critical for speech.

### Dragonfly algorithm

The Dragonfly Algorithm (DA)^25^ is a nature-inspired meta-heuristic optimization technique that was conceptualized based on the static and dynamic collective behaviors of dragonflies. Therefore, it proves very efficient in solving complex optimization problems like feature selection in dimensionality reduction, where a subset of features is chosen that best represents the original dataset. DA allows a manifold reduction that reduces the feature dimensions while retaining all the important information for further use by the successive machine learning model with higher efficiency and improved performance.

#### The DA algorithm operates through the following phases

Initialization: The algorithm initializes the population of dragonflies that are positioned randomly with random velocities in the search space.

The swarming of dragonflies mainly shows two kinds of behavior: static exploration and dynamic exploitation. Exploration spreads out to search for the global optimum, while exploitation focuses its search around the most promising areas.

Objective Function: The fitness of each dragonfly is evaluated by using an objective function, which in dimensionality reduction can be a minimization problem, usually associated with reconstruction error, or a maximization problem associated with variance accounted for.

Position Update: Dragonflies change their positions according to five influencers - dispersion (steering for the avoidance of collision with other dragonflies), alignment (aligning velocity with neighboring dragonflies), cohesion (movement toward the center of a cluster), attraction towards food, and distraction from predators.

It is represented mathematically by Eq.(1).

1 $$\begin{aligned} \textbf{x}_{new} = \textbf{x}_{old} + \alpha \cdot \textbf{r} + \beta \cdot \varvec{\Delta } \end{aligned}$$

where $\textbf{x}_{old}$ and $\textbf{x}_{new}$ are the old and new positions of the dragonfly respectively, $\textbf{r}$ is a random vector, and $\varvec{\Delta }$ is the difference vector between two randomly selected dragonflies. Here $\varvec{\alpha }$ represents the influence of the random vector $\textbf{r}$ controlling the degree of randomness in the movement of the dragonfly. $\varvec{\beta }$ represents the influence of the difference vector $\varvec{\Delta }$, controlling how much the dragonfly’s movement is influenced by the relative positions of other dragonflies.

Iteration: This includes applying the steps iteratively until some convergence criteria, such as a maximum number of iterations or fitness level satisfaction, are satisfied.

**Algorithm 1:**
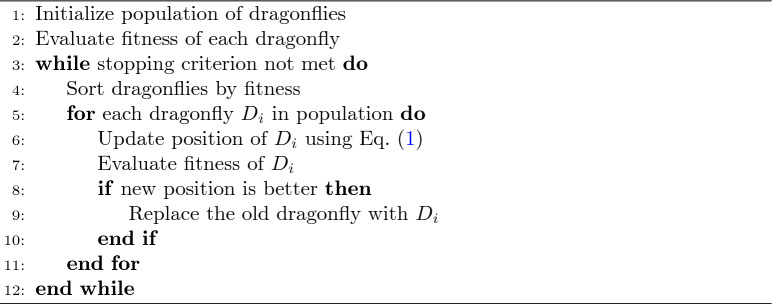
Dragonfly algorithm

### Firefly algorithm

Firefly algorithm (FA)^26^ is one of the optimization techniques that gain inspiration from the flashing actions in fireflies. The technique is mainly effective in solving complex optimization problems and feature selection for reducing dimensions. Reduction in dimensionality using a firefly algorithm may be carried out with no loss to important information, which may improve the efficiency and efficacy of further machine learning models.

#### The FA algorithm operates through the following phases

Attractiveness: The appeal of a firefly is influenced by its luminosity, which correlates with the objective function. More luminous fireflies draw in others, while the level of attractiveness diminishes as distance increases.

Motion: One firefly $(i)$ enchanted by an intra-flying firefly $(j)$ of higher intensity flashes moving towards it is mathematically defined as

2 $$\begin{aligned} X_i = X_i + \beta (X_j - X_i) + \alpha (\rho - 0.5) \end{aligned}$$

where $X_i$ and $X_j$ are the positions of fireflies $i$ and $j$, $\beta$ is the attractiveness parameter, determining how strongly $X_i$ is attracted to $X_j$, $\alpha$ is the randomization parameter, and $\rho$ is a random number between 0 and 1.

**Algorithm 2:**
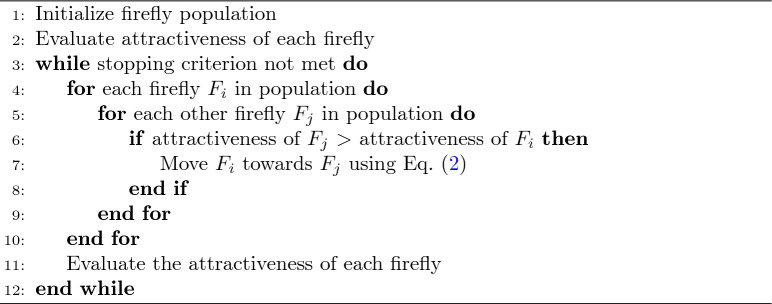
Firefly algorithm

### Moth flame optimization algorithm

The Moth-Flame Optimization (MFO)^27^ probably represents the proposed technique, drawing inspiration from the navigation mechanism practiced by moths; therefore, it could deduce a metaheuristic optimization technique promising to handle complex optimization problems, and more precisely, dimensionality reduction.

#### The MFO algorithm operates through the following phases

Representation of Moth and Flame: MFO represents the solution as moths, and the guiding flames for the moths are the potential solutions. Each moth will get attracted to a flame, simulating the moth navigating toward light.

Position Update: Every moth updates its position concerning every flame since every moth moves toward the flames. Mathematically, the movement of moths can be represented in the following logarithmic spiral equation:

3 $$\begin{aligned} X_i(t+1) = D_i \cdot e^{b \cdot t} \cdot \cos (2 \pi t) + F_j \end{aligned}$$

It means here that $X_i(t+1)$ is the moth position, $D_i$ is the distance between moth and flame, *b* is a constant that defines the shape from a logarithmic spiral, *t* is a random variable in range $[-1, 1]$, and $F_j$ is the flame position.

Flame Update: This reduces the number of flames in every iteration to have a balance between exploration and exploitation.

Therefore, it starts with a high number of flames to explore the search space, and at further steps, the number of flames is reduced for the exploitation phase.

**Algorithm 3:**
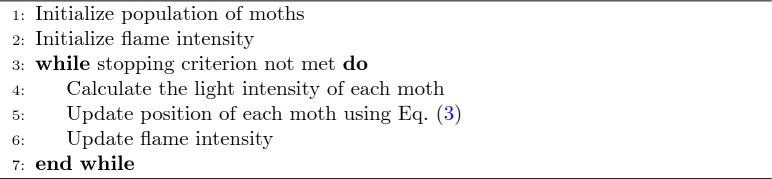
Moth flame optimization algorithm

### System architecture

**Fig. 2:**
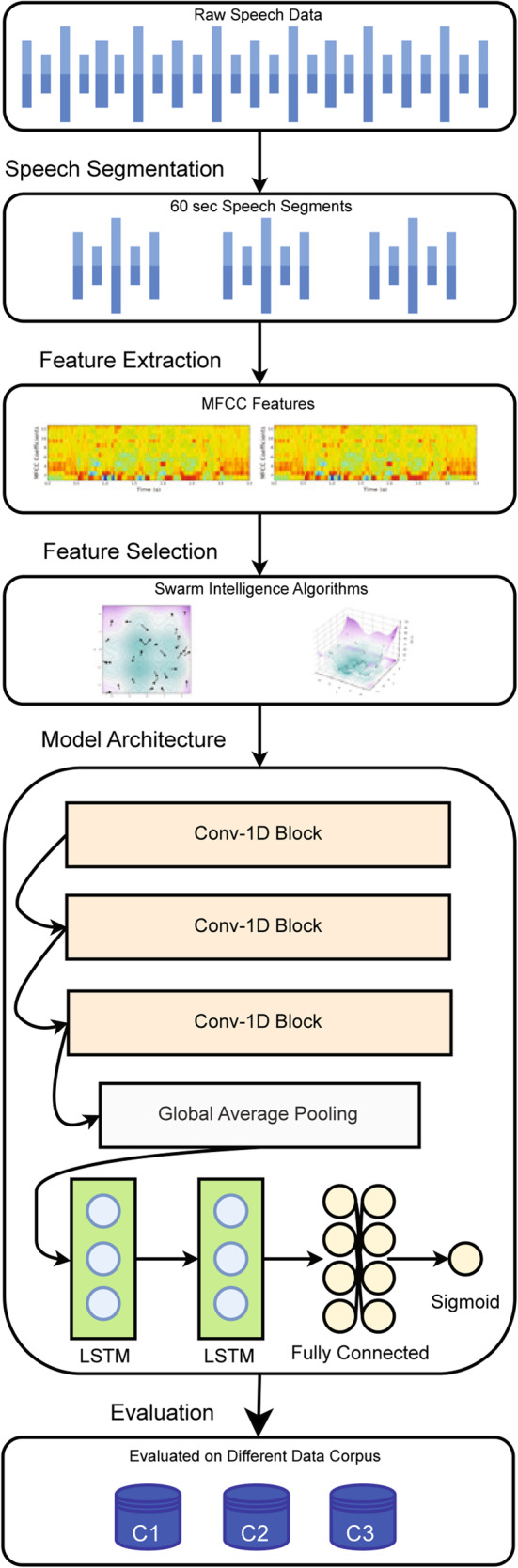
The proposed system architecture.

As shown in Fig. 2, the proposed system architecture starts with feature extraction through MFCC, which captures the essential features of audio. Feature selection will be optimized using swarm intelligence techniques that enhance the performance and reduce the dimensionality. The architecture consists of three 1D convolutional layers with increasing filter sizes, followed by MaxPooling and Dropout for regularization. BatchNormalization is used to stabilize the learning process. Global Average Pooling further reduces dimensionality before feeding the data into two LSTM layers designed for sequence learning. Finally, dense layers are employed for the binary classification task.

### Convolutional layer

The convolutional layer^28^ in CNNs performs the convolutional operation; filters slide over the input data to detect features. Each filter produces a feature map by applying element-wise multiplication and summing up the results. Stride controls the movement of filters, while padding preserves spatial dimensions. To that, one adds non-linearity via an activation function, typically ReLU. This is a critical layer in extracting hierarchical features from input data, used for image classification, object detection, and segmentation. In this way, by learning spatial hierarchies, convolutional layers increase the ability of a model to discover complex patterns and structures from the data.

### Max pooling layer

Max Pooling^28^ involves sliding a window over the input feature map and taking the maximum value from each window. It reduces the feature map size, which in turn reduces computation and hence reduces overfitting. Because this preserves the most dominant features, it helps the network preserve the spatial hierarchies and makes the network more sensitive to recognize patterns and structures in the data. That is really important for stuff like image classification or object detection.

### Dropout layer

Dropout^28^ involves setting a random fraction of the neurons’ output to zero at each training iteration. The effect is that it forces the network to learn redundant representations since it cannot rely on any single neuron. By doing so, Dropout improves the model’s generalization ability and robustness. Normally, one specifies a dropout rate, which indicates how large a portion of dropped neurons should be. This technique works very well, especially for deep networks, since it prevents overfitting and improves their performance on unseen data.

### Long short-term memory

Long Short-Term Memory (LSTM)^29^ networks represent a special variation of RNNs, able to avoid the problem of vanishing gradients that naturally happens with traditional RNNs. This is because the model loses its ability to learn efficiently as the gradients used during training get incredibly tiny. This is resolved by LSTMs, which maintain long-term dependencies in data sequences thanks to their architecture. Three gates-an input gate, an output gate, and a forget gate-and a cell make up an LSTM unit. While gates regulate the flow of information into and out of the cell, the cell must retain the values for a very long time. As a result, this structure enables LSTMs to eliminate irrelevant information and retain important information for a very long time. LSTMs have proven very useful for jobs requiring sequential data, like time series prediction, machine translation, and speech recognition. They also find use in a number of other fields, such as healthcare, robot control, and handwriting recognition.

## Experimental setup

In this section, we explain the setup required to implement the proposed experiments. This setup was meticulously designed to ensure the reliability and validity of the results.

### DIAC WOZ dataset

The Distress Analysis Interview Corpus Wizard-of-Oz^30^ (DAIC-WOZ) dataset is a significant resource in the field of mental health research. It is a sub-project of the greater Distress Analysis Interview Corpus, whose aim goes toward psychological disorders such as anxiety, depression, and PTSD. It is a clinical interview dataset conducted with an animated virtual interviewer they call Ellie, who was controlled by a human interviewer located in another room. The DAIC-WOZ^31^ is composed of 189 individual sessions, each of which ranges from 7 to 33 min, with an average of 16 min per session. The dataset is split into a train set of 107 sessions, a dev set of 35 sessions, and the rest is a test set. Sessions include audio and video recordings, detailed questionnaires, and annotated transcriptions for a myriad of verbal and non-verbal features. As a result, this dataset has become really popular among researchers working on text-based, voice-based, and multimodal detection of depression and other mental health disorders.

### CMDC dataset

The Chinese Multimodal Depression Corpus^32^ (CMDC) dataset consists of semi-structured interviews to support the screening and assessment of major depressive disorder (MDD) in China. These are part of the larger project of creating AI tools capable of interviewing people and detecting the visual, acoustic, and textual signs of MDD. The dataset consists of audio-visual recordings of the interviews, extensive questionnaire responses, and transcriptions annotated for a variety of visual, acoustic, and textual features. There are 78 folders in total, each corresponding to a different subject. All subjects were audio recorded, while only 45 subjects (19 with MDD and 26 healthy controls) were both audio and video recorded.

### MODMA dataset

Multi-modal Open Dataset for Mental-disorder Analysis^33^ (MODMA) dataset designed for mental disorder analysis. Currently, the dataset includes but is not limited to data from clinically depressed patients and matched normal controls, all of whom were carefully diagnosed and selected by professional psychiatrists in hospitals. It is available publicly in two types of data; electroencephalogram (EEG) and speech recording data. For speech data recordings, there were 23 Major Depressive Disorder subjects and 29 Healthy Control subjects. These data were recorded during interviews for people aged 18–52 years old.

### Evaluation criteria

Our depression diagnosis models were compared for their performance using the F1-score, recall, and precision as key metrics. These metrics have been chosen because they comprehensively present the performance of a model, especially when dealing with imbalanced datasets where the number of positive and negative cases can be very different.

- The F1-score is the harmonic mean of precision and recall, providing a single metric that considers both in a balanced way. Because it considers both false positives (FP) and false negatives (FN) defined in Eq. (4), it is particularly useful when the class distribution is uneven.
  4 $$\begin{aligned} {F1-score} = \frac{2*(Recall * Precision)}{Recall + Precision} \end{aligned}$$
- The Recall refers to the ability of the model to identify actual positive cases. It is the ratio of true positives (TP) to the sum of true positives (TP) and false negatives (FN) defined in Eq. (5). High recall suggests that the model is very effective in finding most of the actual positive cases.
  5 $$\begin{aligned} Recall = \frac{TP}{TP+FN} \end{aligned}$$
- Precision is the measure of exactness in the positive predictions of the model. It is defined as the ratio of true positives (TP) to the sum of true positives (TP) and false positives (FP) defined in Eq. (6). High precision means that most of the positive predictions of the model are correct.
  6 $$\begin{aligned} Precision = \frac{TP}{TP+FP} \end{aligned}$$

First, the models were trained and validated on the DAIC-WOZ corpus; after the performance was satisfactory for the DAICWOZ dataset, further evaluation was conducted on two additional datasets CMDC and MODMA to test the generalization capability of the models. These additional evaluations confirm that the models were effective across different data distributions and their reliability in a variety of real-world settings.

### Expermint 1

In this experiment, we designed a deep-learning model for depression diagnosis optimized by the DA. The model was trained and validated on the speech data of the DAIC-WOZ corpus, an increasingly popular dataset for depression detection. It adopted the above system architecture and used the Dragonfly Algorithm for feature selection and dimensionality reduction to ensure maximum performance. The DA parameter settings included a population size of 50 flies with 200 iterations; $\alpha$ was set to 1 initially and decreased linearly to 0.1 throughout the iterations. This strategy allows for strong exploration in the early stages and gradual exploitation toward the end.In which $\beta$ was fixed at 0.5 to maintain a moderate level of social interaction among agents, promoting collaboration while avoiding excessive reliance on neighboring solutions. The model was validated on the development set from DIAC WOZ data, which achieved a 0.76 F1-score, 0.80 recall, and 0.74 precision.

### Expermint 2

In the second experiment, we utilized the already developed system architecture, including the FA for feature selection and dimensionality reduction, to further improve our depression diagnosis model by selecting only the most relevant features and reducing the data complexity. The DAIC-WOZ corpus was again used for training and validation with a large dataset for our analysis. Our previously defined deep neural network architecture was trained using these optimized features and validated on the development set achieving 0.86 F1-score, 0.88 recall, and 0.85 precision. The FA parameter settings included a population size of 50 flies with 200 iterations; $\alpha$ was set to 0.2 , which balances the influence of randomness in the movement of fireflies. A higher $\alpha$ would increase exploration, while a lower $\alpha$ focuses more on exploitation.In which $\beta$ was set to 1.0 , ensuring that the attractiveness of brighter fireflies decreases appropriately with distance. All these metrics show improvement in the model’s accuracy in the diagnosis of depression and hence demonstrate the effectiveness of the Firefly Algorithm in improving feature selection and dimensionality reduction.

### Expermint 3

In the third experiment, feature selection and dimensionality reduction were performed using the MFO algorithm in our system design. It was used to optimize features for improving the performance of our depression diagnosis model. We again trained and validated using the DAIC-WOZ corpus as in our previous experiments. The MFO algorithm was used to choose the most important features and to downsize the dataset to make the model perform well and fast. Our deep neural network architecture, as defined previously, was trained on these optimized features.The MFO parameters were set as a population size of 50 moths with 200 iterations to get the best-fitted features to achieve the performance. The constant *b* was fixed at 1.0, which controls the shape of the logarithmic spiral used to update the position of moths. This ensures a smooth transition between exploration and exploitation. The results achieved were quite encouraging: a 0.80 F1-score, 0.78 recall, and 0.72 precision. These metrics have shown the model to have a good ability in making a correct diagnosis of depression and hence the efficiency of the Moth Flame Optimization Algorithm in enhancing feature selection and dimensionality reduction. This approach shows promise in further improving depression diagnosis models.

## Results and discussion

The results of our experiments reflect the effectiveness and efficiency of the proposed methods for the diagnosis of depression. Each experiment used a different optimization algorithm for feature selection and dimensionality reduction, leading to different performances.

- The first experiment: The method reached a F1-score of 0.76, recall of 0.80, and a precision of 0.74 on the DAIC-WOZ dataset. These are indeed promising results but imply room for further improvements in precision.
- The second experiment: The proposed model showed much improved performance in the Firefly Algorithm, improving its score from the F1-score by 0.86, recall of 0.88, and precision of 0.85, thus confirming that it has supported the dimensionality reduction with appropriate feature selection and improved balanced accuracy of the model.
- The third experiment: After applying this method, a F1-score was achieved at 0.80, the recall was 0.78, and the precision was 0.72. While slightly lower than Firefly, it had a significant uplift in results in comparison to the Dragonfly Algorithm, proving its strengths in feature optimization.

Considering these results with the previous studies that use the same evaluation metrics shown in Table 1 regarding the DAIC-WOZ development set evaluation, our methods yield very good results. Remarkably, the exceptional performance of the FA in our experimental investigations can be linked to several important aspects that strengthen its capability in optimizing the diagnosis of depression based on speech features derived from MFCCs. Firstly, the FA efficiently balances exploration and exploitation via its intrinsic mechanism founded upon attractiveness-driven movement. The attractiveness factor $\beta$ is a monotonically decreasing function of distance, which allows fireflies to roam the whole space globally but gradually focus on good regions as it zooms in toward better solutions. Such a balance is crucial in high-dimensional feature spaces. On the other hand, while the DA relies strongly on social behavior and may occasionally be susceptible to premature convergence if the population size or parameter values are not well-tuned. Similarly, while the MFO is not effective in high-dimensional problems where global exploration is crucial. FA’s stochastic parameter $\alpha$ introduces randomness into the movement of fireflies, enhancing the ability of the algorithm to avoid local optima and explore various regions in the search space. The stochastic component makes FA more robust in handling noisy or uncertain environments compared to DA’s deterministic social behaviors, which limit its ability to adapt to highly dynamic or non-linear issues. The deterministic spiral trajectory of MFO may cause premature convergence in complicated landscapes, particularly if the initial location of moths is not optimal. Furthermore, the features of the FA play a significant role in its enhanced performance in this research, as shown by the high F1-score, recall levels, and precision levels relative to DA and MFO. The fact that FA can efficiently balance exploration and exploitation, resist noisy environments, and preserve population diversity makes it an efficient and powerful algorithm for optimizing the depression diagnostic processes using speech features.

**Table 1:** Comparison between proposed and related models by F1-score, Precision, and Recall on DIAC-WOZ dev-set.

Model	F1-score	Precision	Recall
Zhou et al.^22^	0.77	0.71	0.83
Du et al.^21^	0.74	0.67	0.67
Xu Zhang et al.^20^	0.79	0.84	0.77
Experiment 1	0.76	0.74	0.80
Experiment 2	**0.86**	**0.85**	**0.88**
Experiment 3	0.80	0.72	0.79

Significant values are in bold.

Further experiments with additional two datasets and the evaluation proved the generalizability of our models. In the case of the CMDC dataset, the model achieved a 0.92 F1-score, which indicates excellent performance in another setting. The model achieved a 0.82 F1-score for the MODMA dataset, showing that the performance of the model is consistent and reliable over different datasets. These results validate the proposed methodologies and highlight their potential for improving depression diagnosis using deep learning and optimization algorithms.

## Conclusion and future work

The present study suggested a comprehensive diagnosis scheme by combining the swarm intelligence algorithm with the deep learning architecture. By performing experiments on the DAIC-Woz benchmark dev set, outcomes showed that the Firefly Algorithm outperforms others, which included precision up to 0.85, recall up to 0.88, and F1-score up to 0.86. Dragonfly Algorithm and Moth Flame Optimization Algorithm, with F1-score up to 0.76 and 0.80, respectively, also presented good results. The CMDC and MODMA datasets were used to validate the generalization of these methods further with F1-scores of 0.92 and 0.82, respectively. Notice that the CMDC dataset is population-specific to the Chinese and that the MODMA dataset is a small sample size of 52 subjects. These limitations may affect the extent to which our results can be generalized. Hence, for the generalizability and strength of our method to different populations and cultural settings, additional confirmation in larger and more diverse groups is required. These results indicate the potential of how deep learning and metaheuristic optimization techniques can be combined in terms of accuracy and reliability enhancement for depression diagnosis. The results indicated that the techniques performed better by taking into account the varying distributions of heterogeneous data, leading to enhanced diagnostic performance. Diagnostic performance may potentially be maximized by changing method performance based on the different distributions of heterogeneous data. Even better feature selection and model performance may be obtained by using future research with more advanced optimization techniques. Other data modalities, such as physiological signals and video, may also be utilized to further maximize diagnostic accuracy and knowledge of depression symptoms.

## Data Availability

The datasets used during the current study are available from the corresponding author upon reasonable request. Please get in touch with alwan.atta@cis.asu.edu.eg to know how to access the data.

## References

[1] Institute of Health Metrics and Evaluation. Global Health Data Exchange (GHDx). https://vizhub.healthdata.org/gbd-results/ Accessed 4 December 2024.

[2] Atta, A., Elsayad, D., Ezzat, D., Amin, S. & El Gamal, M. Automated depression screening of clinical transcript optimized by grey wolf optimizer. *Int. J. Intell. Comput. Inf. Sci.* **24**(3), 58–69 (2024).

[3] American Psychiatric Association. Diagnostic and statistical manual of mental disorders 5th ed. (2013).

[4] Evans-Lacko, S. et al. Socio-economic variations in the mental health treatment gap for people with anxiety, mood, and substance use disorders: Results from the WHO World Mental Health (WMH) surveys. *Psychol. Med.* **48**(9), 1560–71 (2018). 29173244 10.1017/S0033291717003336 PMC6878971

[5] Nickson, D. et al. Prediction and diagnosis of depression using machine learning with electronic health records data: A systematic review. *BMC Med. Inform. Decis. Mak* **23**, 271 (2023). 38012655 10.1186/s12911-023-02341-x PMC10680172

[6] Squires, M. et al. Deep learning and machine learning in psychiatry: A survey of current progress in depression detection, diagnosis and treatment. *Brain Inf.* **10**, 10 (2023). 10.1186/s40708-023-00188-6 PMC10123592 37093301

[7] Alsayadi, H., Abdelhamid, A., Hegazy, I. & Taha, Z. Data augmentation for Arabic speech recognition based on end-to-end deep learning. *Int. J. Intell. Comput. Inf. Sci.* **21**(2), 50–64 (2021).

[8] Atta, A., Elsayad, D., Ezzat, D., Amin, S. & El Gamal, M. Speech-based depression detection system optimized using particle swarm optimization. In *The 6th Novel Intelligent and Leading Emerging Sciences Conference* (2024).

[9] Mead, M. Hybrid CNN and LSTM model (HCLM) for short-term traffic volume prediction. *Int. J. Intell. Comput. Inf. Sci.* **22**(4), 51–61 (2022).

[10] Hamed, G., Marey, M., Amin, S. & Tolba, M. Comparative study and analysis of recent computer aided diagnosis systems for masses detection in mammograms. *Int. J. Intell. Comput. Inf. Sci.* **21**(1), 33–48 (2021).

[11] Zhang, et al. Speech-based depression detection using convolutional neural networks. *J. Affect. Disord.* (2023).

[12] Lee, et al. Temporal analysis of speech for depression detection using recurrent neural networks. *IEEE Trans. Neural Netw. Learn. Syst.* (2024).

[13] Kim, et al. Convolutional neural networks for depression detection from speech recordings. *Neurocomputing* (2023).

[14] Wang, et al. Long short-term memory networks for sequential analysis of speech in depression detection. *Pattern Recognit. Lett.* (2024).

[15] Liu, et al. Optimizing convolutional neural networks for depression detection using particle swarm optimization. *Expert Syst. Appl.* (2023).

[16] Gupta, et al. Ant colony optimization for recurrent neural networks in speech-based depression detection. *Appl. Soft Comput.* (2024).

[17] Patel, et al. Hybrid CNN-RNN model optimized by particle swarm optimization for depression detection. *J. Biomed. Inform.* (2023).

[18] Singh, et al. Multimodal depression detection using transformer-based models. *IEEE J. Biomed. Health Informat.* (2024).

[19] Zhao, et al. Explainable AI for speech-based depression detection. *Artif. Intell. Med.* (2023).

[20] Zhang, X., et al. Improving speech depression detection using transfer learning with wav2vec 2.0 in low-resource environments. Scientific Reports (2024). 10.1038/s41598-024-60278-1 PMC11045867 38664511

[21] Du, M. et al. Depression recognition using a proposed speech chain model fusing speech production and perception features. *J. Afect. Disorders.* **323**, 299–308 (2023). 10.1016/j.jad.2022.11.060 36462607

[22] Zhou, Z. et al. Hierarchical multi-feature fusion via audio-response-level modeling for depression detection. *IEEE Trans. Comput. Soc. Syst.* (2022).

[23] Abdul, Z. K. & Al-Talabani, A. K. Mel frequency cepstral coefficient and its applications: A review. *IEEE Access* **10**, 122136–122158 (2022).

[24] Sayf, M., Hafizah, H., Salina, S. & Tarik, I. Mel frequency cepstral coefficients (Mfcc) feature extraction enhancement in the application of speech recognition: A comparison study. *J. Theor. Appl. Inf. Technol.* (2015).

[25] Meraihi, Y. et al. Dragonfly algorithm: A comprehensive review and applications. *Neural Comput. Appl.* **32**, 16625–16646 (2020).

[26] Yang, X. S. Firefly algorithms for multimodal optimization. In *Stochastic Algorithms: Foundations and Applications SAGA 2009 Lecture Notes in Computer Science* Vol. 5792 (eds Watanabe, O. & Zeugmann, T.) (Springer, 2009).

[27] Mirjalili, S. Moth-flame optimization algorithm: A novel nature-inspired heuristic paradigm. Knowl. Based Syst. **89** (2015).

[28] Amin, S. E. & Megeed, M. A. Brain tumor diagnosis systems based on artificial neural networks and segmentation using MRI. In *2012 8th International Conference on Informatics and Systems (INFOS), Giza, Egypt* MM-119-MM-124. (2012).

[29] Atta, A., Elsayad, D., Ezzat, D., Amin, S. & El Gamal, M. Hybrid approach using LSTM neural networks and whale optimization algorithm for enhancing depression diagnosis. In *International Conference on Future Telecommunications and Artificial Intelligence (IC-FTAI)* (2025).

[30] Gratch, J., Artstein, R., Lucas, G. M., Stratou, G., Scherer, S., Nazarian, A., Wood, R., Boberg, J., DeVault, D., Marsella, S. & Traum, D. R. The distress analysis interview corpus of human and computer interviews. In *Proceedings of LREC* (2014).

[31] DeVault, D., Artstein, R. & otehrs. SimSensei kiosk: A virtual human interviewer for healthcare decision support. In *Proceedings of the 13th International Conference on Autonomous Agents and Multiagent Systems (AAMAS ’14)* (2014).

[32] Zou, B. *Chinese Multimodal Depression Corpus* (IEEE Dataport, 2022) .

[33] Hanshu, C., et al. MODMA dataset: A multi-modal open dataset for mental-disorder analysis (2020).

